# Multiparametric Magnetic Resonance Imaging in the Assessment of Primary Brain Tumors Through Radiomic Features: A Metric for Guided Radiation Treatment Planning

**DOI:** 10.7759/cureus.3426

**Published:** 2018-10-08

**Authors:** Edward Florez, Todd Nichols, Ellen E Parker, Seth T. Lirette, Candace M Howard, Ali Fatemi

**Affiliations:** 1 Radiology, University of Mississippi Medical Center, Jackson, USA; 2 Radiation Oncology, University of Mississippi Medical Center, Jackson, USA

**Keywords:** mri multiparametric sequences, intracranial tumors, peritumoral edema, radimic texture features, big data

## Abstract

Purpose

The definition of radiotherapy target volume is a critical step in treatment planning for all tumor sites. Conventional magnetic resonance imaging (MRI) pulse sequences are used for the definition of the gross target volume (GTV) and the contouring of glioblastoma multiforme (GBM) and meningioma. We propose the use of multiparametric MRI combined with radiomic features to improve the texture-based differentiation of tumor from edema for GTV definition and to differentiate vasogenic from tumor cell infiltration edema.

Methods

Twenty-five patients with brain tumor and peritumoral edema (PTE) were assessed. Of the enrolled patients, 17 (63 ± 10 years old, six female and 11 male patients) were diagnosed with GBM and eight (64 ± 14 years old, five female and three male patients) with meningioma. A 3 Tesla (3T) MRI scanner was used to scan patients using a 3D multi-echo Gradient Echo (GRE) sequence. After the acquisition process, two experienced neuroradiologists independently used an in-house semiautomatic algorithm to conduct a segmentation of two regions of interest (ROI; edema and tumor) in all patients using functional MRI sequences, apparent diffusion coefficient (ADC), and dynamic contrast-enhanced MRI (DCE-MRI), as well as anatomical MRI sequences—T1-weighted, T2-weighted and fluid-attenuated inversion recovery (FLAIR). Radiomic (computer-extracted texture) features were extracted from all ROIs through different approaches, including first-, second-, and higher-order statistics, both with and without normalization, leading to the calculation of around 300 different texture parameters for each ROI. Based on the extracted parameters, a least absolute shrinkage and selection operator (LASSO) analysis was used to isolate the parameters that best differentiated edema from tumors while irrelevant parameters were discarded.

Results and conclusions

The parameters chosen by LASSO were used to perform statistical analyses which allowed identification of the variables with the best discriminant ability in all scenarios. Receiver operating characteristic results showcase both the best single discriminator and the discriminant capacity of the model using all variables selected by LASSO. Excellent results were obtained for patients with GBM with all MRI sequences, with and without normalization; a T1-weighted sequence postcontrast (T1W+C) with normalization offered the best tumor classification (area under the curve, AUC > 0.97). For patients with meningioma, a good model of tumor classification was obtained through the T1-weighted sequence (T1W) without normalization (AUC > 0.71). However, there was no agreement between the results of both radiologists for some MRI sequences analyzed for patients with GBM and meningioma. In conclusion, a small subset of radiomic features showed an excellent ability to distinguish edema from tumor tissue through its most discriminating features.

## Introduction

Nearly 80,000 Americans were newly diagnosed with primary brain tumors in 2016, according to the American Brain Tumor Association (ABTA). Of this population, more than 26,000 have primary malignant and 53,000 non-malignant brain tumors [1].

There are essentially two types of brain tumors: primary brain tumors, which begin in the brain and remain within it, and metastatic brain tumors, which begin as cancer in another part of the body and spread to the brain [2-3]. Thus, brain tumors are cataloged as malignant or benign according to their degree of malignancy and aggressiveness, according to guidelines from the World Health Organization (WHO) [3]. However, it is not an easy task to determine if a primary tumor is benign or malignant since various factors, such as pathological characteristics, location, and the type of tissue involved, must be part of this assessment [2,4].

A malignant tumor, also known as brain cancer, is usually rapidly growing, invasive, and potentially deadly. There are four different grades according to the World Health Organization's (WHO’s) grading system [3-5]: (a) grade I tumors grow slowly; their appearance is similar to that of a healthy region, and they are considered non-invasive because their benign tissue composition is unlikely to spread. This grade includes less-malignant tumors associated with long-term survival (e.g. ganglioglioma and gangliocytoma); (b) grade II tumors exhibit relatively slow growth, do not have actively dividing cells, and present a slightly abnormal appearance. Some of these tumors may extend into normal tissue nearby or reappear (e.g. astrocytoma, ependymoma, or oligodendroglioma); (c) grade III tumors are malignant by definition, although the degree of malignancy is based on the cell type since it is not always easy to differentiate these from grade II tumors. Cells from a grade III tumor are obviously abnormal and grow and reproduce actively around normal brain tissue (e.g. anaplastic oligodendroglioma); (d) grade IV tumors divide rapidly, show a distinctly different cellular appearance compared to surrounding normal tissue, have blood vessels to promote their accelerated growth, and have areas of dead tissue in the center (e.g. glioblastoma multiforme).

The two most common types of intracranial tumor are glioblastoma multiforme (GBM) and meningioma [5]. GBM is the most malignant (WHO grade IV) form of intracranial tumor. Its histological characteristics are quite noticeable, with the presence of dead cells (necrotic tissue) and increased blood vessel density around the tumor [3,5-6]. Most cases of meningioma, on the other hand, involve benign tumors, many of which contain calcifications, cysts, or concentrated clusters of blood vessels. However, there are also other types of meningioma such as atypical (10%-15% of meningiomas), and anaplastic (1%-3% of meningiomas) that can also be classified according to the WHO grades [3,7]. Table 1 shows the main characteristics of GBM and meningioma; although both tumor types have distinctive pathophysiologies, they are grouped here because they are considered the most common primary intracranial tumor types encountered in clinical practice [3,8-9].

**Table 1:** Features of glioblastoma and meningioma, two common primary intracranial tumors with a high incidence in adult patients WHO: World Health Organization

Description	Principal Glial Tumor GLIOBLASTOMA	Principal Meningeal Tumor MENINGIOMA
Incidence of occurrence	12% - 20% of all brain tumors	14% - 19% of all brain tumors
Age propensity	45 - 65 years	35 - 70 years
Sex propensity	Almost 2:1 male preponderance^a^	Almost 2:1 female preponderance
Expected locations	Frontal, frontomedial, frontolateral, frontodorsal, temporal, temporomedial including basal ganglia, temporo-parieto-occipital, corpus callosum (butterfly glioma), occipital region, supratentorial	Parasagittal region, falx, convexity, entire skull base, posterior fossa, tentorium, lateral ventricles
WHO grade classification	IV	I, II (atypical) or III (papillary or anaplastic)
Louis et al. [4] report a male-to-female ratio of 1.42:1.		

The term primary brain tumor is usually reserved for an intra-axial tumor originating from normal intracranial cells (astrocytes, oligodendrocytes, etc.). Even meningioma is often not considered a true primary brain tumor because it originates in the extra-axial space.

Once primary brain tumors have been diagnosed, there is a range of different therapy options. The treatment or treatments chosen depend on factors, including the tumor type, its size, its WHO grade, its growth rate, its location in the brain, and the general health of the patient [7]. Surgery, chemotherapy, and radiation therapy (radiotherapy), alone or in combination, are the most common treatments for primary brain tumors [10].

Target volume definition is a critical step in the radiotherapy planning process for all tumor sites. In radiation therapy, precision is key [10-11]. Statistical and image processing techniques may improve the ability to determine the boundaries of tumor and edema. Such improvement will facilitate better tumor control probability (TCP) and limit collateral damage to surrounding normal tissues during treatment. Several studies have been proposed in this regard using different medical modalities (e.g. magnetic resonance imaging (MRI), computed tomography (CT), positron emission tomography (PET), etc.) and diverse approaches (e.g. texture analysis, clustering, support vector machine, machine learning, etc.) [12-13].

The objective of this study was to use MRI images to discriminate tumor from edema and, ultimately, to differentiate different types of edema (e.g. vasogenic and tumor cell infiltration). These techniques involve the use of the radiomic features of multiparametric MRI images to define gross tumor volume (GTV) for treatment purposes.

## Materials and methods

**Figure 1:**
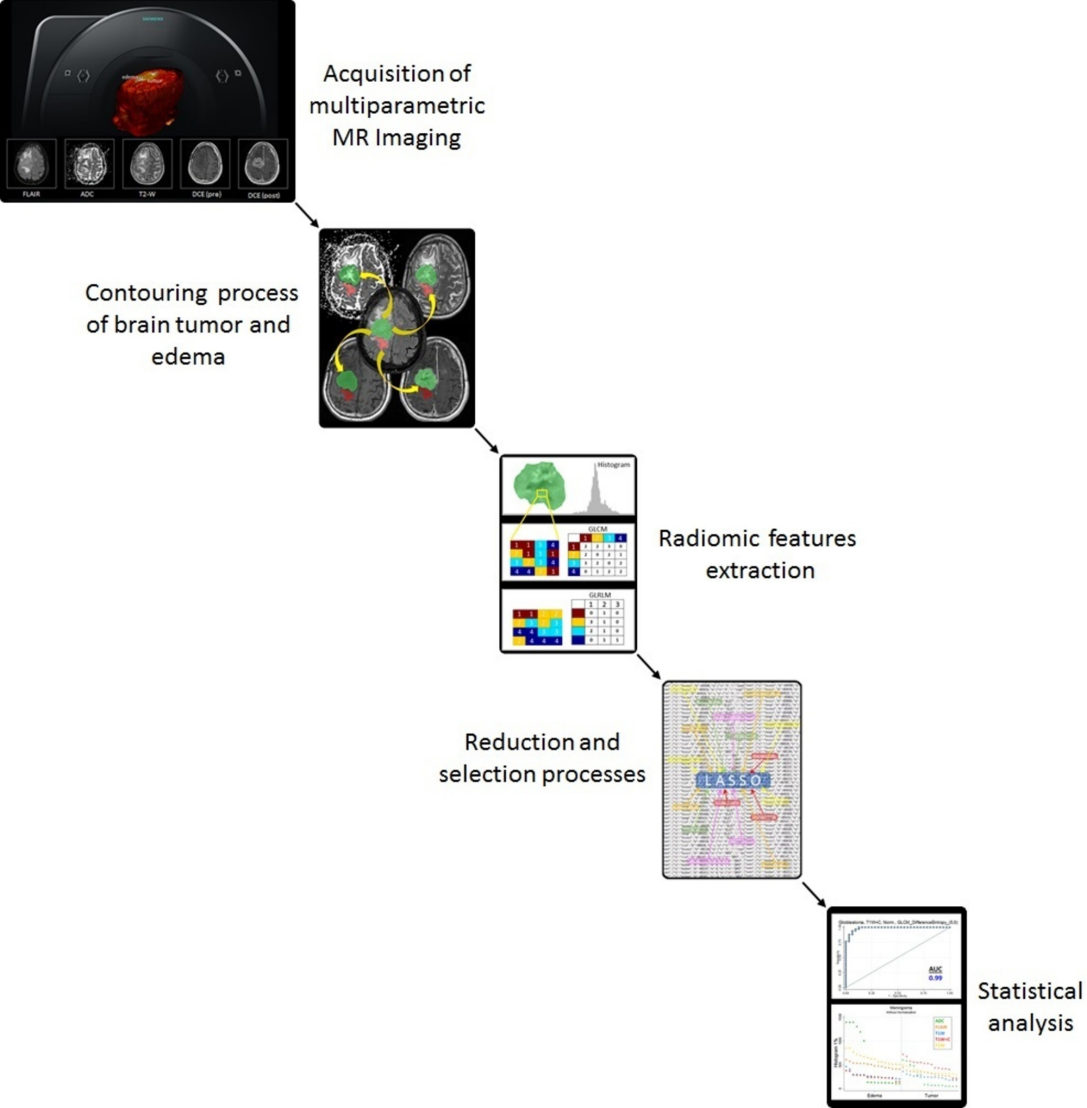
Flow chart with the sequence of modules used in this study: (a) different MRI pulse sequences from patients diagnosed with primary intracranial tumor, (b) contouring process of tumor and edema, (c) radiomic (computer-extracted texture) features extraction, (d) statistical reduction and selection of parameters with the best discriminant ability for distinguishing tumors from edema, and (e) statistical assessment ADC: apparent diffusion coefficient; DCE: dynamic contrast-enhanced; FLAIR: fluid-attenuated inversion recovery; GLCM: gray level co-occurrence matrix; GLRLM: gray-level run-length matrix; LASSO: least absolute shrinkage and selection operator; MRI: magnetic resonance imaging; T2-W: T2-weighted

The study began with the selection of multiparametric, three-dimensional (3D) brain MRI images of patients diagnosed with primary brain tumors. A semi-automatic segmentation process was performed on the functional and anatomical sequences of MRI images by experienced neuroradiologists. They contoured two regions of interest (ROI), edema and tumor, for each enrolled patient. Next, approximately 300 different radiomic (computer-extracted texture) features were pulled from each ROI using different approaches (first-, second-, and higher-order statistics) through in-house MatLab (MathWorks, Inc., Massachusetts, US) codes, with and without normalization. Then, a linear regression statistical method in R, least absolute shrinkage and selector operator (LASSO), was used to reduce and select the parameters that provided the highest association to distinguish tumors from edema while the remaining and irrelevant parameters were eliminated. Finally, a statistical analysis was performed in Stata software (StataCorp., College Station, TX, US) based on the receiver operating characteristic (ROC) curves using the parameters selected by LASSO; relevant figures and tables were constructed based on that analysis. Figure 1 shows a block diagram of the modules developed and used in this study.

Subjects enrolled (study population)

Two experienced neuroradiologists with more than six years of clinical experience selected patients (N = 25) who were diagnosed with primary brain tumors between 2007 and 2016 from among 65 patients of the University of Mississippi Medical Center (UMMC) electronic medical records. Brain tumor and peritumoral edema (PTE) were identified in all enrolled patients.

Of this group, 17 had received diagnoses of glioblastoma (63 ± 10 years old, six female and 11 male patients) and eight had received diagnoses of meningioma (64 ± 14 years old, five female and three male patients).

Inclusion criteria were patient age ≥ 18 years and the presence of pre-operative MRI images with some type of primary cerebral tumor. Exclusion criteria were an incomplete sequence of MRI images (e.g. no non-contrast images), the performance of a preoperative biopsy, and/or patient use of corticosteroids at the time of the preoperative MRI scan. Patients whose files contained MRI images with severe artifacts and excessive noise were also excluded.

Table 2 shows patient demographic information and some important primary characteristics of their brain tumors.

**Table 2:** Patient demographic information and brain tumor characteristics according to the tumor's grade GBM: glioblastoma multiforme; SD: standard deviation; WHO: World Health Organization

	Numbers (%)	WHO grade I	WHO grade II	WHO grade III	WHO grade IV
All patients enrolled	25				
GBM	17 (68)	0	0	0	17
Meningioma	8 (32)	3	3	2	0
Gender					
Male	14 (56)	0	0	0	14
Female	11 (44)	3	3	2	3
Age					
Mean ± SD	65 ± 12	50 ± 11	71 ± 7	77 ± 2	63 ± 10
Tumor Size					
Maximum Length (cm)	4.2 ± 1.2	4.7 ± 0.8	4.2 ± 0.7	3.9 ± 1.1	4.1 ± 1.4
Area (cm^2^)	9.8 ± 5.3	9.5 ± 6.1	12.1 ± 4.2	8.9 ± 5.1	9.5 ± 5.7
Location					
Covexity	7 (28)	0	0	0	7 (28)
Parasagittal	5 (20)	0	0	0	5 (20)
Skull base	3 (12)	0	0	0	3 (12)
Posterior fossa	2 (8)	0	0	0	2 (8)
Frontodorsal	4 (16)	1 (4)	2 (8)	1 (4)	0
Corpus callosum	2 (8)	1 (4)	0	1 (4)	0
Supratentorial	2 (8)	0	2 (8)	0	0

MR imaging (MRI) acquisition

All patients were scanned on a 3 Tesla (3T) scanner (Siemens, Skyra, USA) with a 16-channel radiofrequency (RF) head coil using a T1-weighted contrast-enhanced fast spoiled gradient-recalled acquisition in the steady state; repetition time (TR) = 900 ms, echo time (TE) = 8 ms, matrix size = 256 x 256, slice thickness = 4 mm, bandwidth = 244.141 hertz/pixel, and a T2-weighted (T2-W) fluid-attenuated inversion recovery (FLAIR); TR = 9002 ms, TE = 127.6 ms, inversion time (TI) = 880 ms, matrix = 256 x 256, slice thickness = 5 mm, bandwidth = 122.109 hertz/pixel. MRI scans were acquired prior to radiation treatment.

**Figure 2:**
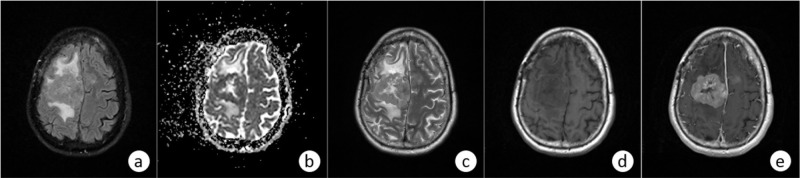
The study used images acquired for each enrolled patient using five different MRI pulse sequences: (a) FLAIR; (b) ADC; (c) T2-W; (d) and (e) DCE pre- and postcontrast, respectively ADC: apparent diffusion coefficient; DCE: dynamic contrast-enhanced; FLAIR: fluid-attenuated inversion recovery; MRI: magnetic resonance imaging; T2-W: T2-weighted

Different MRI sequences images were used in the study (Figure 2), including those acquired with fluid-attenuated inversion recovery (FLAIR), apparent diffusion coefficient (ADC), T2-weighted (T2-W), and dynamic contrast-enhanced (DCE-MRI) techniques. In the case of DCE volumes, pre- and postcontrast images were included. Diffusion-weighted imaging (DWI) and contrast-enhanced (CE) T1-weighted images were obtained in three orthogonal planes after an intravenous injection of 0.1 mmol/kg gadoterate meglumine (Dotarem; Guerbet, Paris, France). Other parameters for the CE T1-weighted image were as follows: section thickness of 3 mm, matrix of 224 × 224, actual voxel size of 1 mm × 1 mm × 1 mm, reconstruction voxel size of 0.5 mm × 0.5 mm × 0.5 mm, number of excitation of 1, SENSE factor of 2, and total acquisition time of 5 minutes and 56 sec. An echo-planar DWI was performed in the axial plane, including image acquisition at b = 0 s/mm2 and diffusion-weighted acquisitions using a standard b = 1000 s/mm2. Other parameters were TR 3000 ms, TE 56 ms, section thickness 5 mm, field of view (FOV) 250 mm, matrix 128 × 128, actual voxel size 1.95 mm × 1.95 mm × 5 mm, reconstruction voxel size 0.98 mm × 0.98 mm × 5 mm, number of excitation = 1, SENSE factor 2.5, and total acquisition time 36 sec. All raw data from DWI were transferred from the MR system to a separate computer for the generation of ADC maps. ADC maps were calculated using b values of 0 s/mm2 and 1000 s/mm2 on a voxel-by-voxel basis.

Image processing

Image processing represents the most important part of this study since the relevant information used from the analysis is extracted from the medical images. All image processing steps were performed using algorithms implemented in MatLab version 2016a, as explained in the following sections. The data processing sequences used in this study allow the analysis of two-dimensional (2D) structures.

Segmentation Process

We measured the signal of edema around GBM and the tumor region in each patient. The same procedure was also repeated in patients with meningioma, using the MRI image sequences described above. Two experienced neuroradiologists independently conducted a segmentation process of the tumor and edema for each patient. The segmentation process was executed manually by each of the neuroradiologists following the same directions at different times.

**Figure 3:**
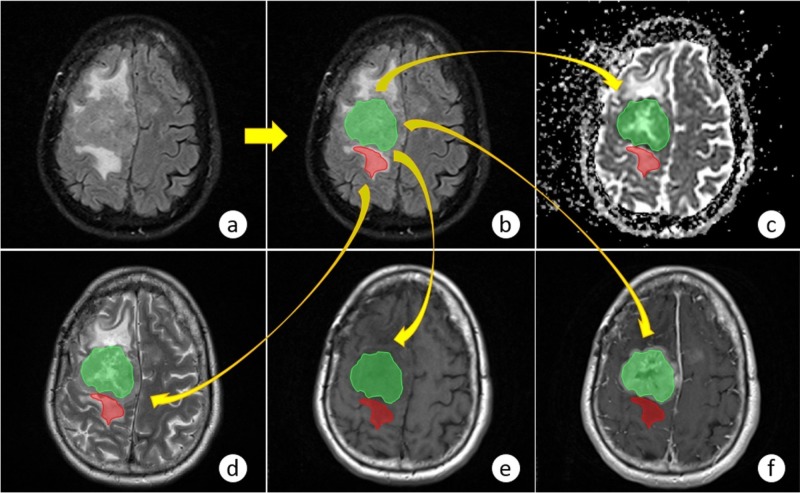
Segmentation process applied to the five different MRI sequences used in the study: (a) original image; (b) FLAIR; (c) ADC; (d) T2-W and DCE; (e) precontrast and (f) postcontrast ADC: apparent diffusion coefficient; DCE: dynamic contrast-enhanced; FLAIR: fluid-attenuated inversion recovery; MRI: magnetic resonance imaging; T2-W: T2-weighted

First, each neuroradiologist performed a previous visual assessment process of all slices that compose the volumes of the images acquired with different MRI sequences. This visual identification allowed the selection of the slice or slices that best represented the ROIs (edema and tumor) in the different images included. For this purpose, all MRI sequences were opened at the same time using OsiriX Lite v.9.0 (Pixmeo, Geneva, Switzerland) [14], a multidimensional digital imaging and communications in medicine (DICOM) visualization tool that allows navigation through all slices of the different volumes in a collective and synchronized way in order to select the correspondent slices to be segmented. Once the required ROIs were contoured in only one MRI sequence, the same segmented areas were replicated in the other sequences through an in-house algorithm implemented in MatLab version 2016a, as shown in Figure 3.

Of the five MRI sequences used in this study, the neuroradiologists reported images acquired with a FLAIR sequence allowed for the easiest identification of both the tumor and PTE regions; thus, the segmentation process was performed on those images in most cases. In some cases, though, it was performed using images acquired using other MRI sequences.

Normalization Process

We performed a normalization process over all segmented ROIs before the extraction of radiomic features. Normalization is a form of image processing that reduces the range of the histogram of each ROI to condense the information for purposes of calculation. We used two approaches to calculate the radiomic features: (a) original ROIs, that is, ROIs without normalization; and (b) ROIs normalized through a method that uses the gray-scale range of the image between 1% and 99% of the cumulated ROI histogram [15]. The 1%-99% normalization process compacts the intensity range of the image by considering the brightness level where the cumulative histogram of the image is between 1% and 99% of its total. In general, the results of normalization processes are typically different for different images; different ROIs regularly yield different 1%-99% levels.

*Radiomic* *Features Extraction*

Radiomic allows the automated extraction of a large number of quantitative characteristics from medical images using advanced feature assessment and data characterization algorithms [16]. This approach allows the quantification of different forms and textures of tumors.

**Figure 4:**
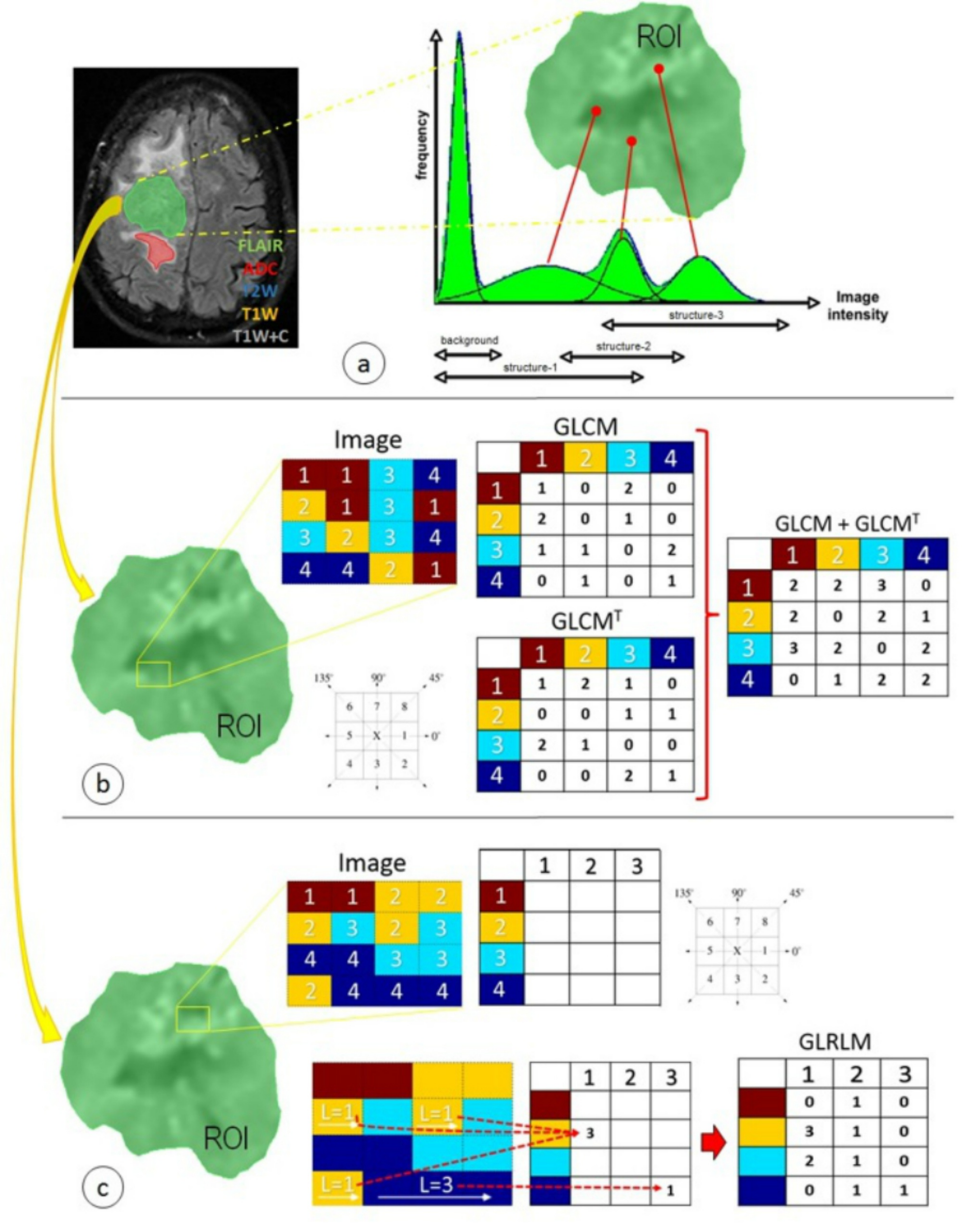
Radiomic features used in this study were distributed in three different techniques focused primarily on statistical approaches: (a) first-order statistics, (b) second-order statistics through the GLCM, and (c) higher-order statistics through the GLRLM ADC: apparent diffusion coefficient; FLAIR: fluid-attenuated inversion recovery; GLCM: gray-level co-occurrence matrix; GLCM^T^: gray-level co-occurrence matrix transpose; GLRLM: gray-level run-length matrix; L: length of homogeneous runs for each grey level; ROI: region of interest; T1W: T1-weighted precontrast; T1W+C: T1-weighted postcontrast; T2W: T2-weighted

Our study performed the extraction of diverse characteristics through an advanced feature analysis. Figure 4 shows three different approaches (first-, second-, and higher-order statistics) used to extract the diverse radiomic features from each of the segmented ROIs, with and without normalization. All approaches were implemented in MatLab version 2016a.

The simplest element constituting a digital image, called pixel or voxel for two-dimensional or three-dimensional images, respectively, is defined by its gray level property, also known as its intensity value [17]. Thus, the gray level distribution of the pixels or voxels of an image can be described through its intensity histogram (Figure 4a). This approach is also referred to as first-order statistics [18] and represents the simplest way of extracting statistical characteristics directly from the digital image. The main advantage of this approach is its simplicity due to the use of standard descriptors to characterize the data. The histogram-based parameters calculated are mean, variance, standard deviation, skewness, kurtosis, energy, entropy, uniformity, coarseness, directionality, contrast, percentiles (1-%, 10-%, 50-%, 90-%, and 99-%), and absolute gradient.

In addition, a second method of extraction of radiomic features, second-order statistics, was implemented. Second-order statistics calculate the probability of a particular relationship between two pixels that have a similarity in a certain gray level at the same time. All information is summarized in a matrix of co-occurrences or correspondences, called the gray level co-occurrence matrix (GLCM), through different distances, d, and different orientations, θ [19]. A relationship of co-occurrence between any two elements can be expressed in general terms as P(i,j,d,θ), where i and j are two neighboring pixel elements with separation distance d and orientation θ. This study examined five different distance values (d = 1, 2, 3, 4, 5) and four different orientations (θ = 0°, 45°, 90°, 135°). The results of the remaining orientations were incorporated through the symmetry property.

Regular and transpose GLCMs were summed by convention in order to obtain a resulting symmetric matrix (Figure 4b) [20]. A set of second-order statistic features were calculated using the symmetric matrix, including autocorrelation, contrast or inertia, correlation, cluster prominence, cluster shade, cluster tendency, dissimilarity, angular second moment (energy or uniformity), entropy, inverse difference moment or homogeneity, inverse variance, maximum probability, sum of squares or variance, sum average, sum variance, sum entropy, difference variance, difference entropy, information measures of correlation, inverse difference-normalized, and inverse difference moment-normalized.

Finally, higher-order statistics were implemented as a third method of extraction of radiomic features. Higher-order statistics [21], an analogous method to second-order statistics, is based on a run-length matrix (Figure 4c) that contains information about the number of runs with pixels of defined grey levels and run lengths in an image, the gray-level run-length matrix (GLRLM) [22]. This matrix was calculated for different run angles (θ = 0°, 45°, 90°, 135°), and a set of higher-order statistics features were calculated using the GLRLM, such as short-run emphasis, long-run emphasis, gray level non-uniformity, run length non-uniformity, run percentage, low gray level run emphasis, high gray level run emphasis, short-run low gray level emphasis, short-run high gray level emphasis, long-run low gray level emphasis, long-run high gray level emphasis, gray level variance, and run length variance.

Equations for the statistical characteristics mentioned in this section can be found in the supplementary information (appendixes) of our previous study [23].

Statistical assessment

Agreement between the two neuroradiologists’ classification of tumor versus edema was assessed by Kappa statistics. In fact, the two neuroradiologists agreed 100% of the time, even across the five different MR imaging sequence types. The texture analysis method applied in this study has a statistical basis [24], and its values were computed for our specific population of ROIs: edema and intracranial tumor, both with and without normalization. The statistical assessment was the result of an estimation process of a large database formed by all texture parameters previously calculated using the least absolute shrinkage and selection operator (LASSO) [25]. Binomial families, logit links, and linear regression models were used to reduce and select the parameters that provide the highest association for distinguishing tumors from edema while shrinking irrelevant parameters.

Principal component analysis (PCA) and/or Fisher scoring are the standard operating procedures for doing a variable selection on groups of variables in linear regression models [26]. However, both standard methods are unable to classify the variable of interest (edema versus tumor, in this particular study) thus not efficiently identifying the desired ROIs.

LASSO is more consistent and allows us to construct a model specifically tailored to classify tumor versus edema. One additional issue that arises with this particular study is that there is no unified statistical framework to account for the variability between readers for the LASSO selection. For example, there is not an established procedure for penalized regression approaches to select variables across multiple readers. The set of variables (texture parameters) selected to best classify tumor versus edema likely will vary across readers. To this end, we independently ran LASSO procedures on each of the two neuroradiologists’ data and only considered texture parameters selected in both. The regularization parameter λ was chosen via cross-validation as the λ that minimized the prediction error.

While over-fitting could be a potential issue with a sample size this small, we believe nested cross-validation is unwarranted in this situation [27]. By choosing only the parameters chosen by LASSO from both readers, we are being more conservative than choosing a model based upon the lowest cross-validated Brier score or highest cross-validated area under the curve (AUC). Using these selected parameters, a receiver operating characteristic (ROC) analysis was performed and all relevant plots were constructed. The LASSO procedure was performed in R using the glmnet package and all other statistical procedures were performed using Stata v14.1 [28].

## Results

Table 3 shows the set of variables selected for each scenario through the LASSO procedure using both expert neuroradiologists’ readings. The number of variables chosen varied depending only on the different scenarios (GBM and meningioma, both with and without normalization); the maximum number of variables selected for some scenarios was four. In some meningioma scenarios, certain thresholds were sufficient for the perfect classification of tumor and edema. Take, for example, the non-normalized FLAIR sequence for meningioma. In this scenario, we found that histogram percentile 90% < 520 perfectly classified tumor from edema as shown below.

**Table 3:** Set of parameters selected by LASSO procedure involving two expert neuroradiologists. The selection includes the radiomic features with the best discriminant ability to differentiate edema from tumor tissue for different MRI sequences, different primary tumoral disease and different scenarios ADC: apparent diffusion coefficient; d: distance between the pixel of interest and its neighbor; FLAIR: fluid-attenuated inversion recovery; GLCM: gray-level co-occurrence matrix; GLRLM: gray-level run-length matrix; LASSO: least absolute shrinkage and selection operator; MRI: magnetic resonance imaging; NA: no agreement; T1W: T1-weighted precontrast; T1W+C: T1-weighted postcontrast; T2W: T2-weighted

		Sequence	Parameter(s)	Most Useful Parameter*
Meningioma	Non-normalized	ADC	GLCM Correlation, 90°, d = 1; GLCM Sum Average, 90°, d = 1; GLCM Sum Average, 45°, d = 1; Histogram Skewness	GLCM Sum Average, 90°, d = 1; GLCM Sum Average, 45°, d = 1^b^
		FLAIR	Histogram Percentile 90%	Histogram Percentile 90%^d^
		T1W	GLRLM Gray-Level Non-Uniformity 0°; Histogram Skewness	Histogram Skewness
		T1W+C	GLCM Difference Entropy, 135°, d = 4; Histogram Percentile 99%	Histogram Percentile 99%^e^
		T2W	Histogram Percentile 1%; Histogram Percentile 50%; Histogram Skewness	Histogram Percentile 1%; Histogram Percentile 50%^f^
	Normalized	ADC	None Selected^c^	NA^c^
		FLAIR	None Selected^c^	NA^c^
		T1W	None Selected^c^	NA^c^
		T1W+C	Histogram Percentile 99%	Histogram Percentile 99%^e^
		T2W	GLCM Sum Average, 135°, d = 5; GLRLM Gray-Level Non-Uniformity 90°; Histogram Percentile 1%; Histogram Percentile 50%	Histogram Percentile 1%; Histogram Percentile 50%^f^
Glioblastoma	Non-normalized	ADC	GLCM Entropy, 135°, d = 5	GLCM Entropy, 135°, d = 5
		FLAIR	None Selected^c^	NA^c^
		T1W	None Selected^c^	NA^c^
		T1W+C	GLCM Correlation, 135°, d = 2	GLCM Correlation, 135°, d = 2
		T2W	GLCM Entropy, 135°, d = 5; Histogram Kurtosis; Histogram Percentile 99%	GLCM Entropy, 135°, d = 5
	Normalized	ADC	GLCM Difference Entropy, 135°, d = 5; GLCM Sum Variance, 90°, d = 3	GLCM Difference Entropy, 135°, d = 5
		FLAIR	GLCM Sum Average, 45°, d = 5; GLCM entropy, 0°, d = 1; Histogram Percentile 1%	GLCM Entropy, 0°, d = 1
		T1W	Absolute Gradient Skewness	Absolute Gradient Skewness
		T1W+C	Absolute Gradient Skewness; GLCM Difference Entropy, 0°, d = 5; Histogram Percentile 99%	GLCM Difference Entropy, 0°, d = 5
		T2W	GLCM Entropy, 135°, d = 5	GLCM Entropy, 135°, d = 5
Defined as a parameter with the best ability to discriminate tumor from edema within each tumor type				
GLCM Sum Average, 90°, d = 1 or GLCM Sum Average, 45°, d = 1 < 130 perfectly classifies tumor for meningioma, non-normalized ADC sequence				
No agreement between readers for LASSO results				
Histogram Percentile 90% < 520 perfectly classifies tumor for meningioma, non-normalized FLAIR sequence				
Histogram Percentile 99% > 600 perfectly classifies tumor for meningioma, non-normalized, or normalized T1W+C sequence				
Histogram Percentile 1% < 500 or Histogram Percentile 50% < 600 perfectly classifies tumor for meningioma, non-normalized, or normalized T2W sequence				

LASSO selected the radiomic texture features with the best ability to discriminate tumor from edema for the different scenarios, considering the different sequences of MRI images. However, a few scenarios were also reported in which no parameters were selected (marked as None Selected or NA in Table 3).

AUC values for the most useful parameters in discriminating tumor from edema in patients diagnosed with GBM and meningioma are shown in Table 4 and Table 5, respectively. The AUC results show that all univariate models had a good discriminant ability to classify tumor from edema in patients with GMB in all scenarios and MRI sequences for which a discriminant parameter was selected. The T1-weighted sequence postcontrast (T1W+C) with normalization was the model with the best tumor classification (AUC > 0.97).

**Table 4:** AUCs for the most useful parameter for classifying tumors in patients diagnosed with GBM ADC: apparent diffusion coefficient; AUC: area under the curve; CI: confidence interval; d: distance between the pixel of interest and its neighbor; FLAIR: fluid-attenuated inversion recovery; GBM: glioblastoma multiforme; GLCM: gray level co-occurrence matrix; NA: no agreement; T1W: T1-weighted precontrast; T1W+C: T1-weighted postcontrast; T2W: T2-weighted

Sequence		Parameter With Best Discriminant Ability	AUC (95% CI)
Non-normalized	ADC	GLCM entropy, 135°, d = 5	0.91 (0.85-0.98)
	FLAIR	None Selected^a^	NA^a^
	T1W	None Selected^a^	NA^a^
	T1W+C	GLCM Correlation, 135°, d = 2	0.85 (0.75-0.94)
	T2W	GLCM Entropy, 135°, d = 5	0.91 (0.83-0.98)
Normalized	ADC	GLCM Difference Entropy, 135°, d = 5	0.90 (0.81-0.98)
	FLAIR	GLCM Entropy, 0°, d = 1	0.82 (0.73-0.92)
	T1W	Absolute Gradient Skewness	0.69 (0.56-0.82)
	T1W+C	GLCM Difference Entropy, 0°, d = 5	0.99 (0.97-1.00)
	T2W	GLCM Entropy, 135°, d = 5	0.85 (0.75-0.94)
No agreement between readers for LASSO results			

**Table 5:** Areas under the curve (AUCs) for the most useful parameter for classifying tumors in patients diagnosed with meningioma ADC: apparent diffusion coefficient; AUC: area under the curve; CI: confidence interval; d: distance between the pixel of interest and its neighbor; FLAIR: fluid-attenuated inversion recovery; GLCM: gray level co-occurrence matrix; NA: no agreement; PC: perfect classification; T1W: T1-weighted precontrast; T1W+C: T1-weighted postcontrast; T2W: T2-weighted

Sequence		Parameter With Best Discriminant Ability	AUC (95% CI)
Non-normalized	ADC	GLCM Sum Average, 0°, d = 1 & GLCM Sum Average, 45°, d = 1 < 130	PC^b^
	FLAIR	Histogram 90% < 520	PC^b^
	T1W	Histogram skewness	0.85 (0.71-0.99)
	T1W+C	Histogram 99% > 600	PC^b^
	T2W	Histogram 1% & Histogram 50% < 500	PC^b^
Normalized	ADC	None Selected^a^	NA^a^
	FLAIR	None Selected^a^	NA^a^
	T1W	None Selected^a^	NA^a^
	T1W+C	Histogram 99% > 600	PC^b^
	T2W	Histogram 1% & Histogram 50% < 500	PC^b^
No agreement between readers for LASSO results			
Perfect classification given conditions of the third column			

A good model of tumor classification for patients with meningioma was obtained from images obtained with a T1-weighted sequence without normalization (AUC > 0.71). However, there was no agreement by both neuroradiologists for meningioma when using other MRI sequences. Consequently, it was not possible to obtain AUC results for those cases.

**Figure 5:**
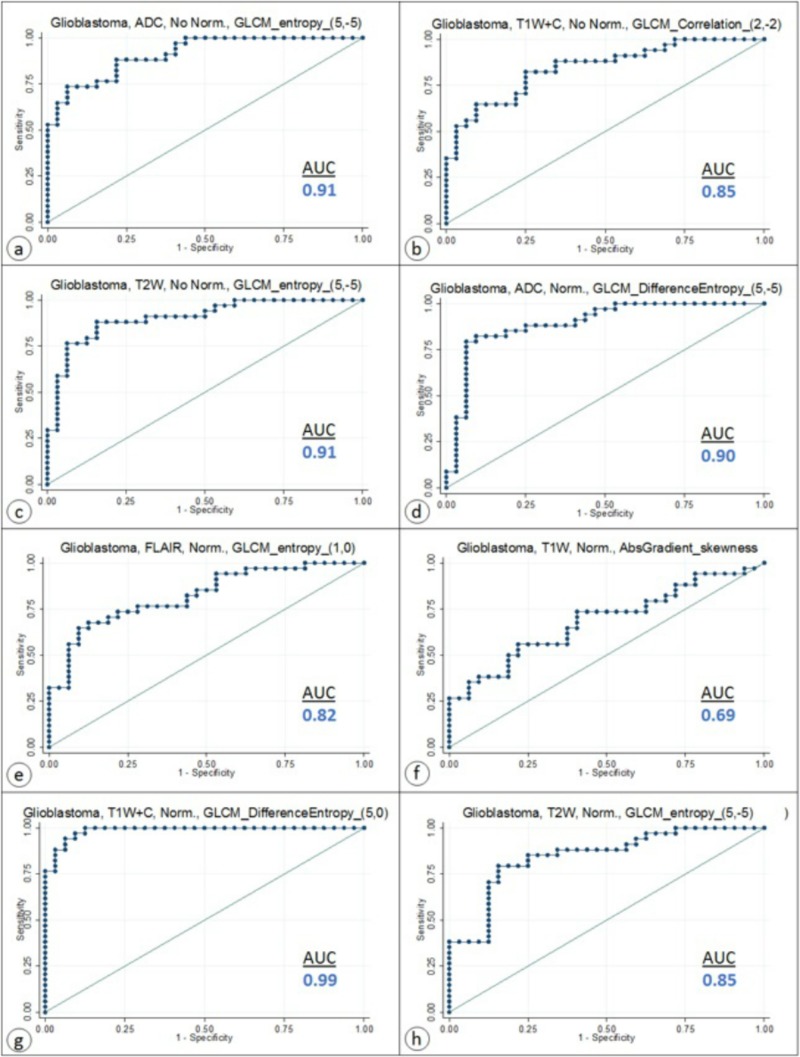
AUCs for the most useful parameter for discriminating tumors in patients diagnosed with GBM using (a)-(c) MRI sequences without normalization and (d)-(h) MRI sequences with normalization. Some scenarios have no AUC value since no parameters were chosen by LASSO ADC: apparent diffusion coefficient; AUC: area under the curve; FLAIR: fluid-attenuated inversion recovery; GBM: glioblastoma multiforme; GLCM: gray level co-occurrence matrix; LASSO: least absolute shrinkage and selection operator; MRI: magnetic resonance imaging; T1W: T1-weighted precontrast; T1W+C: T1-weighted postcontrast; T2W: T2-weighted

Additionally, AUC values for the most useful parameters for discriminating edema from tumor in patients diagnosed with GBM can be seen in Figure 5. Some scenarios are presented without an AUC value since no parameters were chosen by LASSO.

**Figure 6:**
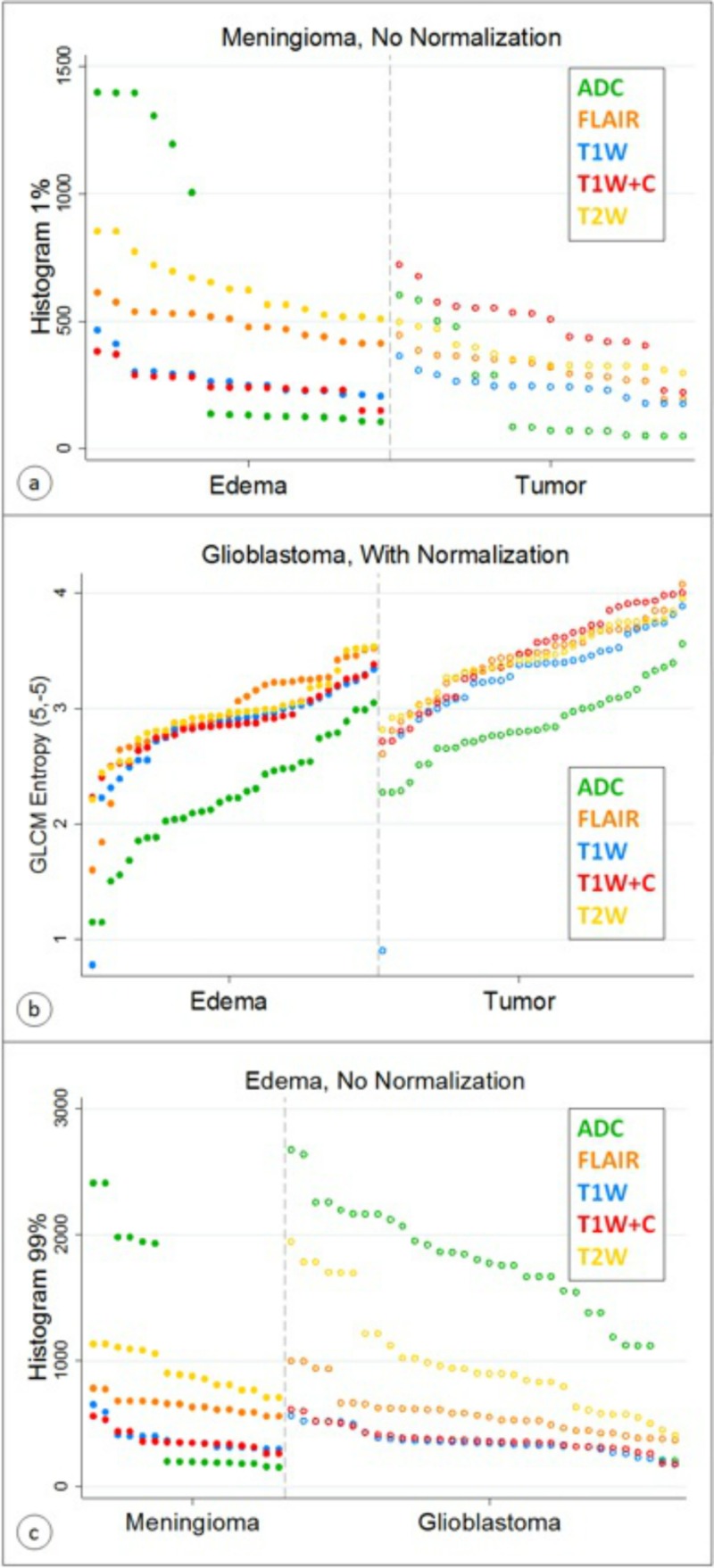
Sorted values for the best discriminator stratified by tissue (edema and tumor) for different MRI sequences in patients with (a) meningioma without normalization, (b) GBM with normalization, and (c) for specific types of edema linked to patients diagnosed with meningioma and GBM ADC: apparent diffusion coefficient; FLAIR: fluid-attenuated inversion recovery; GBM: glioblastoma multiforme; GLCM: gray level co-occurrence matrix; MRI: magnetic resonance imaging; T1W: T1-weighted precontrast; T1W+C: T1-weighted postcontrast; T2W: T2-weighted

Finally, the discriminant capacity of differentiating both ROIs in patients diagnosed with meningioma and GBM could be exemplified with the best parameters selected by LASSO. Figures 6a-6b show the sorted values for the best discriminator stratified by tissue (edema and primary brain tumors) for images acquired with different MRI sequences in patients with each tumor type. Also, through the same MRI sequences, the sorted values for the best discriminator stratified for specific types of edema linked to patients diagnosed with meningioma and GBM, are shown in Figure 6c.

## Discussion

Ten out of every 100,000 people in the United States are diagnosed with primary brain tumors: glioblastoma multiform (GBM) or meningioma [1]. The risk of intracranial tumors can be elevated by environmental factors, such as infections, hereditary factors, hormonal factors, high-risk encephalo-cranial traumas, and exposure to radiation sources, among others [2,5,8]. Although there are a considerable number of anatomopathological variables, it is necessary to have an adequate protocol through which a histological diagnosis can be established in order to increase the odds of success of treatment. The effective differentiation and delineation of the target volume will suggest better tumor control probability (TCP) and limit collateral damage to surrounding normal tissues from treatment.

Fortunately, a series of significant developments have taken place in recent years, providing better tools to treat brain tumors. However, the success of these treatments depends directly on accuracy in estimating the location and size of the brain tumor.

In this clinical study, we assessed the ability of radiomic (computer-extracted texture) features to distinguish edema from primary brain tumors in images acquired using a variety of functional and anatomical MRI sequences. The texture analysis was capable of generating relevant information that can serve radiologists in the organization, diagnosis, and characterization of lesions or types of tumors in the brain. Well-known radiomic concepts and different texture analysis approaches, such as first-, second-, and higher-order statistics, were used for this purpose.

A small number of radiomic texture features was selected from a set of several hundred parameters initially calculated through distinct scenarios, different MRI sequences, and diverse approaches. Those selected parameters allowed the segregation of the tumor regions in the brain and the differentiation of edema and tumor tissue.

The reduction and selection processes are commonly executed through techniques such as PCA, Fisher scoring, and linear discriminant analysis (LDA). This study used a different selection operator, LASSO, for the selection of the representative parameters that provide the highest association for distinguishing tumors from edema while simultaneously shrinking irrelevant parameters.

There is a clear differentiation not only of location, size, and shape but also of biological and morphological characteristics between the two types of tumors analyzed in this study. For example, GBM is considered a solitary tumor, usually located in the frontal lobes of the brain. This study not only showed the differentiation between the two different brain pathologies evaluated (meningioma and GBM) but also suggests a differentiation between the edema around both intracranial tumors. Edema surrounding GBM is known to contain both vasogenic and tumor cell infiltration while edema surrounding meningioma is a vasogenic type without tumor cell infiltration [6].

According to the most useful selected descriptors and their theoretical definitions, the radiomic texture features that describe patients with meningioma are mostly first-order parameters obtained directly from the histogram of the images acquired using different MRI sequences, which suggests a similarity and linear dependency between the intensity values of its elements (pixels, in our case). Analogously, the radiomic texture features that describe patients with GBM are mostly second-order parameters obtained through a particular relationship (probability) between two pixels that have similarity in a certain gray level at the same time. These statements should be taken with caution since they refer only to the cases analyzed in this study and cannot yet be generalized.

Finally, our preliminary results appear to suggest that radiomic (computer-extracted texture) features may provide important diagnostic information based on images obtained using routine MRI pulse sequences. This information may improve the distinction of edema from tumor for primary brain tumors, but these results need to be validated with larger sample sizes.

Study limitations and future directions

Despite the interesting use of radiomics in a clinical application, the main limitation of this study is the small number of patients enrolled (N = 25). While radiomic features have the proven potential to discriminate edema from tumor tissue, and our processing structure is well-developed and considers all the basic components of radiomic, different procedures and tools could be incorporated and tested in future studies to potentially offer improved results.

Therefore, our future efforts will be oriented to add specific transformation mechanisms (fast Fourier transform (FFT), S-transform, Hartley transform, etc.) on the input multiparametric MRI images. In addition, this study examined the normalization process of the ROIs based on 1%-99% normalization, where the chosen features are shown to be dependent on the normalization of the image. However, approaches such as the limitation of dynamics to µ ± 3σ (where µ is the mean gray-level value and σ is the standard deviation), and gray level compression based on the range between δ and 2δ (where δ is the number of bits per pixel), among others, can also be tested and compared. These alternative normalization methods have been used in recent clinical studies by Collewet and coworkers [29], among others. Likewise, our future work in this area will be focused on the extraction of the texture characteristics of 3D volumes of interest instead of just 2D regions of interest. We expect that this will optimize the accuracy of tumor segmentation and maximize the results of therapeutic procedures planned using this technique. A recent clinical study conducted by Ortiz-Ramon and collaborators [30] shows the superiority of using 3D texture features with interesting results (AUC > 0.9). We will also prospectively validate the radiomic texture features identified in this study in a larger and multi-institutional cohort, both in the context of differentiating edema from brain tumor and with other regions such as radiation necrosis, among others.

## Conclusions

Radiomic (computer-extracted texture) features offer a remarkable quantitative approach that allows the representation, differentiation, and characterization of diverse regions within the same medical image through appropriate descriptors. Of several hundred radiomic texture features extracted from ROIs belonging to enrolled patients, only a few parameters selected by a statistical operator were necessary to perform a clear discrimination of edema from tumor tissue.
